# The effect of timing of decompression on neurologic recovery and histopathologic findings after spinal cord compression in a rat model

**Published:** 2012-11

**Authors:** Seyed Behzad Jazayeri, Masoumeh Firouzi, Shayan Abdollah Zadegan, Niloufar Saeedi, Elham Pirouz, Mohsen Nategh, Issa Jahanzad, Ahmad Mohebbi Ashtiani, Atefeh Yazdi, Mehrbod Karimi, Vafa Rahimi-Movaghar

**Affiliations:** ^a^Sina Trauma and Surgery Research Center, Tehran University of Medical Sciences, Tehran, Iran.; ^*b*^Student’s Scientific Research Center, Tehran University of Medical Sciences, Tehran, Iran.; ^*c*^Tissue Repair Laboratory, Institute of Biochemistry and Biophysics (IBB), University of Tehran, Tehran, Iran.; ^*d*^Research Centre for Neural Repair, University of Tehran, Tehran, Iran.; ^*e*^Department of Pathology, Faculty of Medicine, Tehran University of Medical Sciences, Tehran, Iran.; ^*f*^Department of Neurosurgery, Zahedan University of Medical Sciences , Zahedan, Iran.

## Abstract

**Background:**

To examine whether spinal cord decompression improves functional recovery and decreases lesion volumes in paraplegic rats two studies were conducted. At the first study, a comparative study was conducted to evaluate decompression timing of 3-seconds, 1 hour, 6 hours, 3 weeks and 10 weeks in 63 rats. The second study was performed to compare the electro-microscopic findings and motor function recovery in a group of 10 rats receiving compression for 3 seconds and 10 minutes.

**Methods:**

Compressive injury was produced using an aneurysmal clip method. Behavioral assessment was performed through inclined plane test, tail-flick reflex and Basso, Beattie and Bresnahan (BBB) rating scales. At the end of the study, the rats were sacrificed and spinal cord specimens were studied under light and electron microscopic techniques.

**Results:**

The first experiment showed that rats sustaining 3-second immediate spinal cord compression showed signiﬁcant improved functional recovery (P less than 0.05) and smaller lesion volumes (P = 0.039) than rats subjected to compression times of 1 hour, 6 hours, 3 weeks, and 10 weeks after spinal cord injury. BBB rating scales were significantly better in the early compression group after the 4th week of evaluation (P less than 0.05) and persisted throughout the rest of the study. Histopathology demonstrated decreased normal tissue, more severe gliosis and cystic formation in the late group, compared to the early group (P less than 0.05). In EM study, injuries in the late group including injury to the myelin and axon were more severe than the early compression group and there was more cytoplasmic edema in the late compression group.

**Conclusions:**

Our findings show that the early 3-second decompression after spinal cord injury improves functional motor recovery, spares more functional tissue, which is accompanied by lower intracellular edema and myelin and axon damage, whereas higher myelin regeneration in rats compared to those with 10 minutes of compression. Inclined plane and tail-flick reflex assessments showed no significant difference.

**Keywords:**

Decompression, BBB rating scal , Histopathology, Electron microscopy, Spinal cord injury


					Volume 4, Suppl. 1 Nov 2012
Publisher: Kermanshah University of Medical Sciences
URL: http://www.jivresearch.org
Copyright:
Congress of Iranian Neurosurgeons, 14-16 November 2012, Kermanshah, Iran
Paper No. 86
The effect of timing of decompression on neurologic recovery
and histopathologic findings after spinal cord compression in
a rat model
Seyed Behzad Jazayeri a,b
, Masoumeh Firouzi c
, Shayan Abdollah Zadegan a
, Niloufar Saeedi a
,
Elham Pirouz a
, Mohsen Nategh d
, Issa Jahanzad e
, Ahmad Mohebbi Ashtiani d
, Atefeh Yazdi f
,
Mehrbod Karimi f
, Vafa Rahimi-Movaghar a, d,*
a
Sina Trauma and Surgery Research Center, Tehran University of Medical Sciences, Tehran, Iran.
b
Student's Scientific Research Center, Tehran University of Medical Sciences, Tehran, Iran.
c
Tissue Repair Laboratory, Institute of Biochemistry and Biophysics (IBB), University of Tehran, Tehran, Iran.
d
Research Centre for Neural Repair, University of Tehran, Tehran, Iran.
e
Department of Pathology, Faculty of Medicine, Tehran University of Medical Sciences, Tehran, Iran.
f
Department of Neurosurgery, Zahedan University of Medical Sciences , Zahedan, Iran.
Abstract:
Background: To examine whether spinal cord decompression improves functional recovery and decreases lesion
volumes in paraplegic rats two studies were conducted. At the first study, a comparative study was conducted to
evaluate decompression timing of 3-seconds, 1 hour, 6 hours, 3 weeks and 10 weeks in 63 rats. The second study
was performed to compare the electro-microscopic findings and motor function recovery in a group of 10 rats
receiving compression for 3 seconds and 10 minutes.
Methods: Compressive injury was produced using an aneurysmal clip method. Behavioral assessment was performed
through inclined plane test, tail-flick reflex and Basso, Beattie and Bresnahan (BBB) rating scales. At the end of the
study, the rats were sacrificed and spinal cord specimens were studied under light and electron microscopic
techniques.
Results: The first experiment showed that rats sustaining 3-second immediate spinal cord compression showed
significant improved functional recovery (P less than 0.05) and smaller lesion volumes (P = 0.039) than rats subjected
to compression times of 1 hour, 6 hours, 3 weeks, and 10 weeks after spinal cord injury. BBB rating scales were
significantly better in the early compression group after the 4th week of evaluation (P less than 0.05) and persisted
throughout the rest of the study. Histopathology demonstrated decreased normal tissue, more severe gliosis and
cystic formation in the late group, compared to the early group (P less than 0.05). In EM study, injuries in the late
group including injury to the myelin and axon were more severe than the early compression group and there was
more cytoplasmic edema in the late compression group.
Conclusions: Our findings show that the early 3-second decompression after spinal cord injury improves functional
motor recovery, spares more functional tissue, which is accompanied by lower intracellular edema and myelin and
axon damage, whereas higher myelin regeneration in rats compared to those with 10 minutes of compression.
Inclined plane and tail-flick reflex assessments showed no significant difference.
Keywords:
Decompression, BBB rating scal , Histopathology, Electron microscopy, Spinal cord injury
* Corresponding Author at:
Vafa Rahimi-Movaghar: Associate professor of Neurosurgery, Research Deputy, Sina Trauma and Surgery Research Center, Sina Hospital, Hassan-
Abad Square, Imam Khomeini Ave, Tehran University of Medical Sciences, Tehran, Iran. Phone: (+98) 915 342 2682, (+98) 216 6757010,
Fax: (+98) 216 675 7009, Email: v_rahimi@sina.tums.ac.ir , v_rahimi@yahoo.com, (Rahimi-Movaghar V.).

				

